# Changes in rat plasma proteomes during the first week after birth

**DOI:** 10.3389/fvets.2025.1440716

**Published:** 2025-02-25

**Authors:** Lilong Wei, Zheng-guang Guo, Jing Wei, Yun Zhou, Wei Sun, Chengwu Han

**Affiliations:** ^1^Clinical Laboratory Center, China-Japan Friendship Hospital, Beijing, China; ^2^Core Facility of Instruments, School of Basic Medicine, Peking Union Medical College, Institute of Basic Medical Sciences, Chinese Academy of Medical Sciences, Beijing, China; ^3^Department of Pharmacy, Clinical Research Center, National Center for Children's Health, Beijing Children's Hospital, Capital Medical University, Beijing, China

**Keywords:** plasma proteome, development, LC-MS/MS, label-free quantification, organ development

## Abstract

Blood plasma is the most informative body fluid, containing large amounts of substances that are released by active secretion or leakage from tissues and cells. Therefore, plasma changes reflect the body state. To explore changes in plasma during the early life of Wistar-rats, the plasma proteomes of newborn and first-week rats were investigated using liquid chromatography—tandem mass spectrometry. A total of 639 proteins were identified at both developmental stages and 570 proteins were used for quantitative analysis. The plasma of first-week rats, compared to that in newborn rats using label-free quantification, showed that the levels of 42 proteins significantly increased while those of 17 proteins decreased. Plasma proteomic patterns at both developmental stages can be easily separated using differential protein cluster analysis. Using the Ingenuity Pathway analysis tool, some pathways including LXR/RXR Activation, DHCR24 Signaling Pathway, Acute Phase Response Signaling, and Detoxification of Reactive Oxygen Species were significantly enhanced. Over 10 categories related to the development and functions were enriched. Plasma proteomes of first-week rats were distinct from those of newborn rats. These changes would make it easier for newborn rats to survive. This is the first study using liquid chromatography—tandem mass spectrometry to investigate newborn rat plasma proteome changes, providing a basis and clues for studying animal development.

## 1 Introduction

Blood plasma is one of the most complex and informative fluids, that contains numerous proteins and other substances (1, 2). As blood plasma flows through almost all tissues or organs of the body and contains various released substances, it is one of the best reporter systems in clinical settings (3, 4). Hundreds of proteins have been identified using little serum or plasma based on the development of proteomics technologies (5). Profiling can provide further information on various diseases, physiological processes, individualization, and diversity (6–8).

The birth process is a distinct physiological event, and newborns exhibit unique life characteristics. After birth, neonates rapidly change in early life owing to complicated biological processes. The morphological changes of early life have been described in detail (9, 10). During development, each organ undergoes different changes and simultaneously works with others to perform delicate biological functions (11–13). It is worthwhile analyzing the age-related differences in the plasma proteome, and it is recognized as a scientific aim and research priority by The Human Proteome Organization Plasma Proteome Project (37). Significant changes were observed from newborns and children to adults using two-dimensional difference gel electrophoresis (2D-DIGE) (4). Additionally, serum samples from individuals since birth until 36 months of age were studied using label-free quantitative proteomic analyses (14). The blood plasma of neonates, children, and adults was identified and quantified, and protein expression variations were displayed (3). The plasma proteomic profile study of 10 healthy children during development was analyzed using TMT-10plex isobaric labeling-based quantitative proteomics (15). The plasma proteome and immune system were reported to change in the first week of human life (16). To better show the experimental information of the relevant literature, we have summarized a simple table (Table 1). These studies revealed age-specific protein profiles in humans. However, our knowledge of animal development during early life is limited. Although there may be similarities in development among different species, it is still necessary to describe the early life development of animals objectively and in detail to enhance human understanding of animals and facilitate related animal work. Rat is widely used in human medical research as important animal models for human diseases. The serum proteins of rats from the earliest suitable developmental stage to newborn was analyzed using paper electrophoresis (17). To better understand the changes in animal plasma after birth, the plasma proteomes of newborn (NB) rats and rats 1 week after birth were compared using the label-free quantitative proteomics technology of liquid mass spectrometry. This is the first time that liquid mass spectrometry proteomics has been used to study plasma changes after birth in rats. The results from this study may provide further information on this complex biological fluid during growth and development in rats.

**Table 1:** Proteomic studies of serum or plasma during postnatal development.

**Time period**	**Species**	**Number of samples**	**Screening criteria**	**Number of proteins differentially expressed**	**Experimental technique**	**Reference**
Neonate to adult	Human	6	*P* ≤ 0.05 Foldchange >1.5	40 proteins with 100 proteins spots	Two-dimensional difference in gel electrophoresis (2D-DIGE)	(4)
Neonate to adult	Human	10	*P* ≤ 0.05 Foldchange >1.5	107	Liquid chromatography mass spectrometry (LC-MS/MS)	(3)
9 months to 15 years of age	Human	10	*P* ≤ 0.05	100 to 266 protein groups with different trends	Isobaric labeling-based LC-MS/MS	(15)
Neonate to 36 months	Human	15	Percentage of false prediction < 0.05	122	LC-MS/MS	(14)
Neonate to 7days	Human	10–30	5% FDR with BH correction and +/− 0.2 log2 foldchange	96	LC-MS/MS	(16)

## 2 Materials and methods

### 2.1 Animal experiments

This study was performed according to the ethical conditions stated in the International Animal Welfare Recommendations and was approved by the Animal Ethics Committee of the China-Japan Friendship Hospital (Animal ethics permission number 180108). Pregnant Wistar rats (250 g) were purchased from Beijing Wei-tong-li-hua Laboratory Animal Technology Company (Beijing, China). Blood of NB rats and first-week (FW) rats was collected from the jugular vein (17). The first drop of blood was not collected to avoid potential contamination. Blood was immediately mixed with ethylenediaminetetraacetic acid-K2 (1.5 g/L). Samples were centrifugated at 1,000 g for 15 min to remove the blood cells. After centrifugation at 12,000 g, the supernatant was stored at −80°C (18).

### 2.2 Mass spectrometry (MS) analysis

#### 2.2.1 Sample preparation

Rat plasma samples were digested as described in the literature (19). The protein samples to be digested were diluted four times with 25 mM NH4HCO3, vortexed, and mixed for 1 min. Then 1M DTT was added to make the final concentration 20 mM in the solution and incubated in a 95°C water bath for 5 min. The samples were removed and allowed to cool to room temperature, and then 1M IAA was added to make the final concentration 50 mM in the solution. The samples were treated in the dark for 45 min and centrifuged at 12,000 g for 10 min. A new 30KD filtration membrane was taken out, and 100 ul UA was added to the filtration tube of the 30K membrane. The lid was covered and centrifuged at 14,000 g for 1 min, repeated 2–3 times. The upper layer was slowly aspirated into the 30K filtration tube treated with UA. The 10KD filter membrane was placed with added protein sample in a centrifugal machine and centrifugal for 10–30 min at 14,000 g; after the sample was fully filtered, 200 ul UA was added to the inner sleeve, vortexed for 1 min, then centrifugal for 10–30 min at 14,000 g; 200 ul 25 mM NH_4_HCO_3_ was added to the inner sleeve of the filter tube, vortexed for 1 min and centrifugal for 15–30 min at 14,000 g; 200 ul 25 mM NH_4_HCO_3_ as added to the inner sleeve of the filter tube, and Trypsin enzyme was added (according to the protein: Trypsin enzyme = 50:1 mass ratio), vortexed for 1 min; the sample with trypsin was fixed on a water float, placed the water ticket on the water surface, put in the microwave oven, high fire for 1 min, and finally the sample was put in the beaker into a water bath pot, water bath at 37°C for 16–24 h; taken out, centrifugated for 1–10 min at 14,000 g, 50–100 uL 500 mM NaCl was added and beated repeatedly, centrifugal for 1–10min at 14,000 g, the liquid was aspirated into a new EP tube, then desalted using an Oasis HLB cartridge (Waters, Milford, MA, USA).dry in a vacuum dryer, and dissolved with one-thousand FA after drying (the dissolution concentration is generally 5 ug/ul).

### 2.3 Liquid chromatography–MS analysis

Plasma peptides were loaded onto an offline high-pH reversed-phase LC column (4.6 mm × 250 mm, C18, 3 μm; Waters, Milford, MA, USA) in buffer A1 (H_2_O, pH 10) and eluted with 5–30% buffer B1 (90% acetonitrile, pH 10; flow rate 0.7 mL/min) for 30 min. Individual samples were analyzed in DIA mode according to a previously described DIA-MS workflow.

Mass spectrometry analysis was performed using an Orbitrap Fusion Lumos MS (Thermo Scientific, Dreieich, Germany) with an EASY-nLC 1000 LC system. The digested peptides were separated using an RP C18 self-packed capillary LC column and analyzed in DDA mode for generating the spectral library (20).

For the DIA analysis, 21 variable separation windows were used for MS acquisition. The full scan range was set from 350 to 1,200 m/z with a screening resolution of 120,000, followed by a DIA scan with a resolution of 30,000 (HCD collision energy: 32%; Automatic Gain Control target: 1,000,000; maximum injection time: 50 ms).

### 2.4 Data analysis

Proteome Discoverer (Thermo Scientific, Dreieich, Germany) was used to process the DDA data. Then the data were searched against the rat UniProtFASTA database (version 2017_09) with added indexed Retention Time fusion protein sequences. Two missed tryptic digestion cleavage sites were allowed for searching with the parameter settings as previously described. Spectronaut Pulsar software (Biognosys, Schlieren, Switzerland) was used to generate spectral libraries and analyze the DIA-MS raw files. Proteins with at least one unique peptides were identified at an FDR threshold of 1% at the protein level (20). The data were processed in the following manner: Proteins with more than 50% missing values in both groups were discarded. The minimum value was used to fill in the missing values for the remaining proteins. If a protein is not identified in more than 50% of the six samples at a specific time point, the minimum value from all identified instances of that protein in the experiment, including both experimental samples and the mixed serum from the two groups, was used for imputation. For proteins with 50% or fewer missing values, we applied the K-nearest neighbor method for imputation using the wukong platform (https://www.omicsolution.org/wkomic/main/).

### 2.5 Statistical analysis

In the experiment, the protein abundance was log2 normalized, and then T-test was used to find differentially expressed proteins. The FDR were controlled by applying the Benjamini–Hochberg correction method (21). Proteins with fold change >2 and p.adjust.BH < 0.05 were considered as significant differential proteins. Principal component analysis and Orthogonal Partial Least Squares Discriminant Analysis were performed on the identified proteins to demonstrate the differences between the two time points in neonatal rats using the wukong platform (https://www.omicsolution.org/wkomic/main/). A volcano plot was mapped to express the changed proteins based on the screening criteria. Hierarchical clustering was performed to identify protein clusters. The ROC curve analyses were performed using MetaboAnalyst 6.0 software for significant differential proteins (https://www.metaboanalyst.ca/MetaboAnalyst).

### 2.6 Bioinformatics analysis

All changed proteins between the two different developmental stages were assigned gene symbols for functional annotation assessment by the Database for Annotation, Visualization, and Integrated Discovery (22). In ingenuity pathway analysis (IPA), the UniProt accession numbers of the proteins were uploaded to IPA software (QIAGEN, Germanland, MD, USA). Disease and function categories and canonical pathways of the proteins were analyzed and ranked according to their *p*-value. The enrichment significance of each function and network was calculated using a one-sided Fisher's exact test. DAVID Functional Annotation online tool was used for functional analysis (https://davidbioinformatics.nih.gov/).

## 3 Results

### 3.1 Comparison of plasma proteomics between FW and NB rats

In our study, the plasma of FW and NB rats were used for comparative analyses. Six individual plasma samples from FW and NB rats were analyzed using LC-MS/MS. Using the criterion of 1% FDR levels for both the peptide and protein, 639 proteins were identified in all MS runs with at least one unique peptide and 570 proteins were used for quantitative analysis. In the plasma proteome of FW rats compared to that of NB rats, 59 proteins showed significant changes in abundance with fold change >2 and p.adjust.BH < 0.05 (Supplementary Table S1), and the top 10 significantly changed proteins (Fold change >10) are shown in Table 2. The proteomic data from similar human studies were compared. The significant proteins identified in our study and those reported previously are listed in Table 3.

**Table 2:** Significantly differentiated proteins (fold change > 10) in plasma proteome of FW rats vs. NB rats.

**Uniprot accession**	**Gene names**	**Protein names**	**p.adjust.BH**	**Fold change**	**AUC**
P35577	Serpina7 Tbg	Thyroxine-binding globulin	0.0023749	26.27	1
P23593	Apod	Apolipoprotein D	0.0101759	24.97	1
Q62840	Ambn	Ameloblastin	0.005244	18.51	1
P10959	Ces1c Es2	Carboxylesterase 1C	0.0285319	16.44	0.94
Q9Z339	Gsto1	Glutathione S-transferase omega-1	0.0453962	16.17	0.94
P55797	Apoc4 Ecl	Apolipoprotein C-IV	0.0227943	12.74	0.97
P55314	C8b	Complement component C8 beta chain	0.0458831	11.91	0.94
P05371	Clu	Clusterin	0.016198	11.37	1
Q8K3U6	F7	Coagulation factor VII	0.0181774	10.71	1
P31211	Serpina6 Cbg	Corticosteroid-binding globulin	0.0023749	0.10	1

**Table 3:** Comparations of the results in our study to those obtained priory in similar studies in human plasma proteomics.

**Uniprot accession**	**Gene name**	**Protein names**	**Fold change**	**Our result**	**Published result**	**Reference**
P35577	Serpina7 Tbg	Thyroxine-binding globulin	26.27	↑	↑	(3)
P23593	Apod	Apolipoprotein D	24.97	↑	↑	(3)
P05371	Clu	Clusterin	11.37	↑	↑	(3, 4)
P02651	Apoa4	Apolipoprotein A-IV	9.05	↑	↑	(3)
Q62930	C9	Complement component C9	7.87	↑	↑	(3, 16)
Q9EQV8	Cpn1	Carboxypeptidase N catalytic chain	7.79	↑	↑	(3)
P01015	Agt Serpina8	Angiotensinogen	6.91	↑	↓	(3, 16)
P20759		Ig gamma-1 chain C region	6.41	↑	↑	(3)
Q7TMA5	Apob Aa1064 Ac1-060	Apolipoprotein B-100	5.59	↑	↑	(4)
P02466	Col1a2	Collagen alpha-2(I)	5.19	↑	↑	(3)
P08649	C4 C4a	Complement C4	4.53	↑	↑	(3)
P14272	Klkb1 Klk3 Pk	Plasma kallikrein	3.94	↑	↑	(3, 4)
P11517		Hemoglobin subunit beta-2	0.29	↓	↓	(3, 4)
P06866	Hp Ba1-647	Haptoglobin	0.28	↓	↑	(3, 16)
P21743	Igfbp1 Igfbp-1	Insulin-like growth factor-binding protein 1	0.26	↓	↑↓	(3, 16)

**Figure 1:**
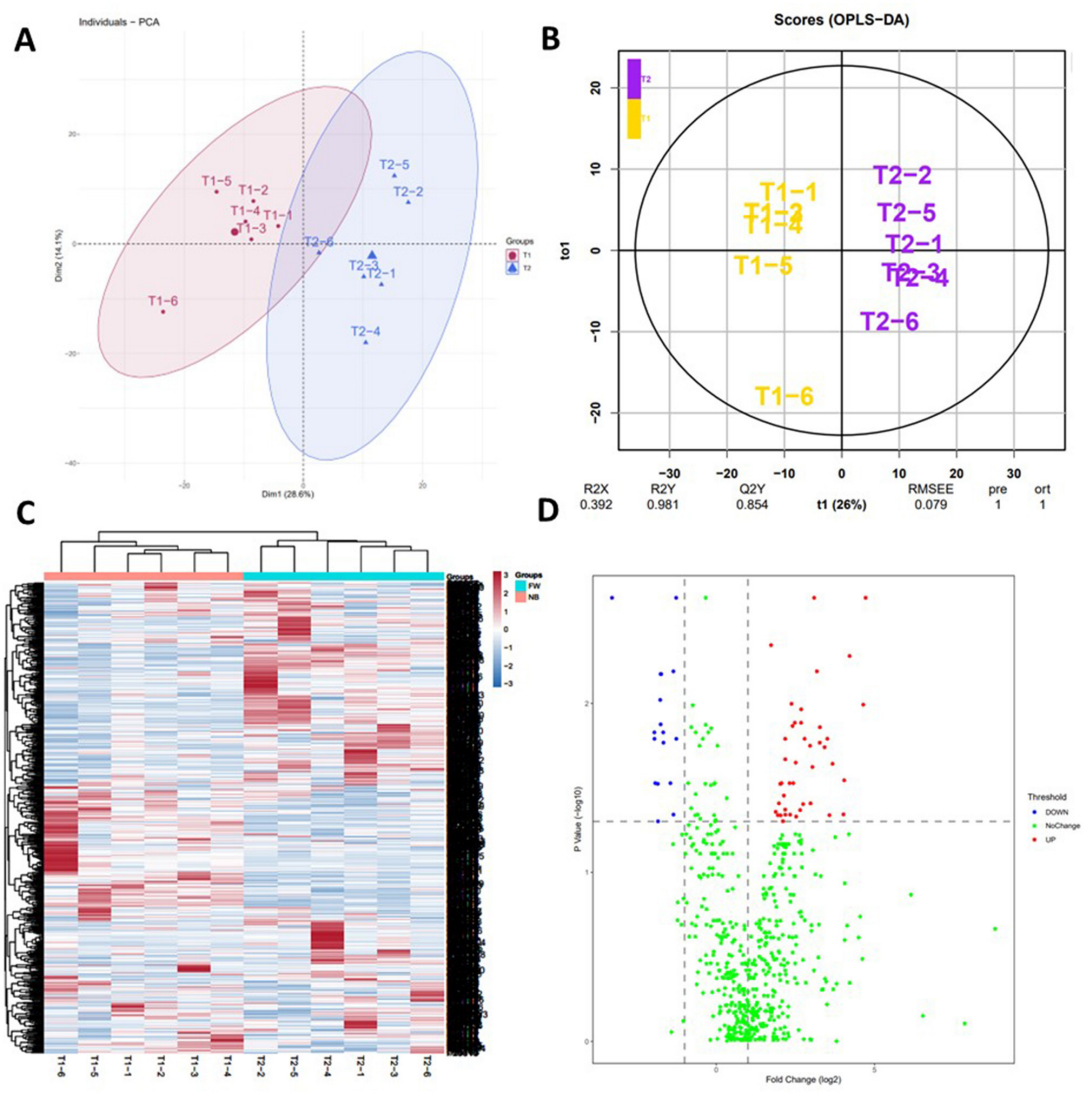
Overall distribution of identified proteins. **(A)** Principal component analysis and Orthogonal partial least squares discriminant analysis of the identified proteins in plasma proteomics. **(B)** Orthogonal partial least squares discriminant analysis of the identified proteins in plasma proteomics. **(C)** Hierarchical clustering analysis of the all identified proteins in the two stage samples. In the color bar, red represents high levels, and blue represents low levels [**(B)** Blue—proteins at low levels; Red—proteins with high levels; T1 stands for NB samples, and T2 stands for FW samples]. **(D)** Significantly changed proteins analyzed by volcano plots between two stage samples [**(A)** FW rats vs. NB rats].

Principal component analysis and Orthogonal partial least squares discriminant analysis of the identified proteins in plasma proteomics showed that plasma proteomics could well distinguish the two groups of samples (Figures 1A, B). To better show the differences in development among different individuals, all identified proteins were analyzed by clustering (Figure 1C). The two-stage samples can be easily distinguished from the map. Simultaneously, it was interesting to note that there were obvious differences in the same group, we speculated that there were individual differences in the development of different rat. A volcano plot was used to show the significantly expressed proteins screened using the above criteria (Figure 1D). In mass spectrometry analysis, equal amounts of protein were loaded, and the protein intensity values were normalized in quantitative analysis. When the volcano plot analysis was performed, a rightward shift was observed, which may be due to the increasing trend of more proteins.

### 3.2 Function enrichment of Gene Ontology (GO) categories analysis

The significantly changed proteins were functionally classified according to the general GO annotation terms (23). In the biological process analysis, the top five terms were hydrogen peroxide catabolic process (GO:0042744), cellular oxidant detoxification (GO:0098869), positive regulation of CoA-transferase activity (GO:1905920), lipid transport (GO:0006869), and removal of superoxide radicals (GO:0019430) (Table 4).

**Table 4:** Top 10 terms classified using general GO annotation terms.

**Category**	**Term**	**Count**	**%**	***P* value**
GOTERM_BP_DIRECT	GO:0042744~hydrogen peroxide catabolic process	7	11.11	2.7E-10
GOTERM_BP_DIRECT	GO:0098869~cellular oxidant detoxification	8	12.70	3.1E-09
GOTERM_BP_DIRECT	GO:1905920~positive regulation of CoA-transferase activity	4	6.35	1.5E-06
GOTERM_BP_DIRECT	GO:0006869~lipid transport	6	9.52	1.7E-05
GOTERM_BP_DIRECT	GO:0019430~removal of superoxide radicals	4	6.35	1.8E-05
GOTERM_BP_DIRECT	GO:0042542~response to hydrogen peroxide	5	7.94	0.00011
GOTERM_BP_DIRECT	GO:0097421~liver regeneration	5	7.94	0.00014
GOTERM_BP_DIRECT	GO:0042246~tissue regeneration	4	6.35	0.00016
GOTERM_BP_DIRECT	GO:0006958~complement activation, classical pathway	4	6.35	0.00018
GOTERM_BP_DIRECT	GO:0009410~response to xenobiotic stimulus	9	14.29	0.00028

### 3.3 Gene networks of ingenuity pathway analysis (IPA)

**Figure 2:**
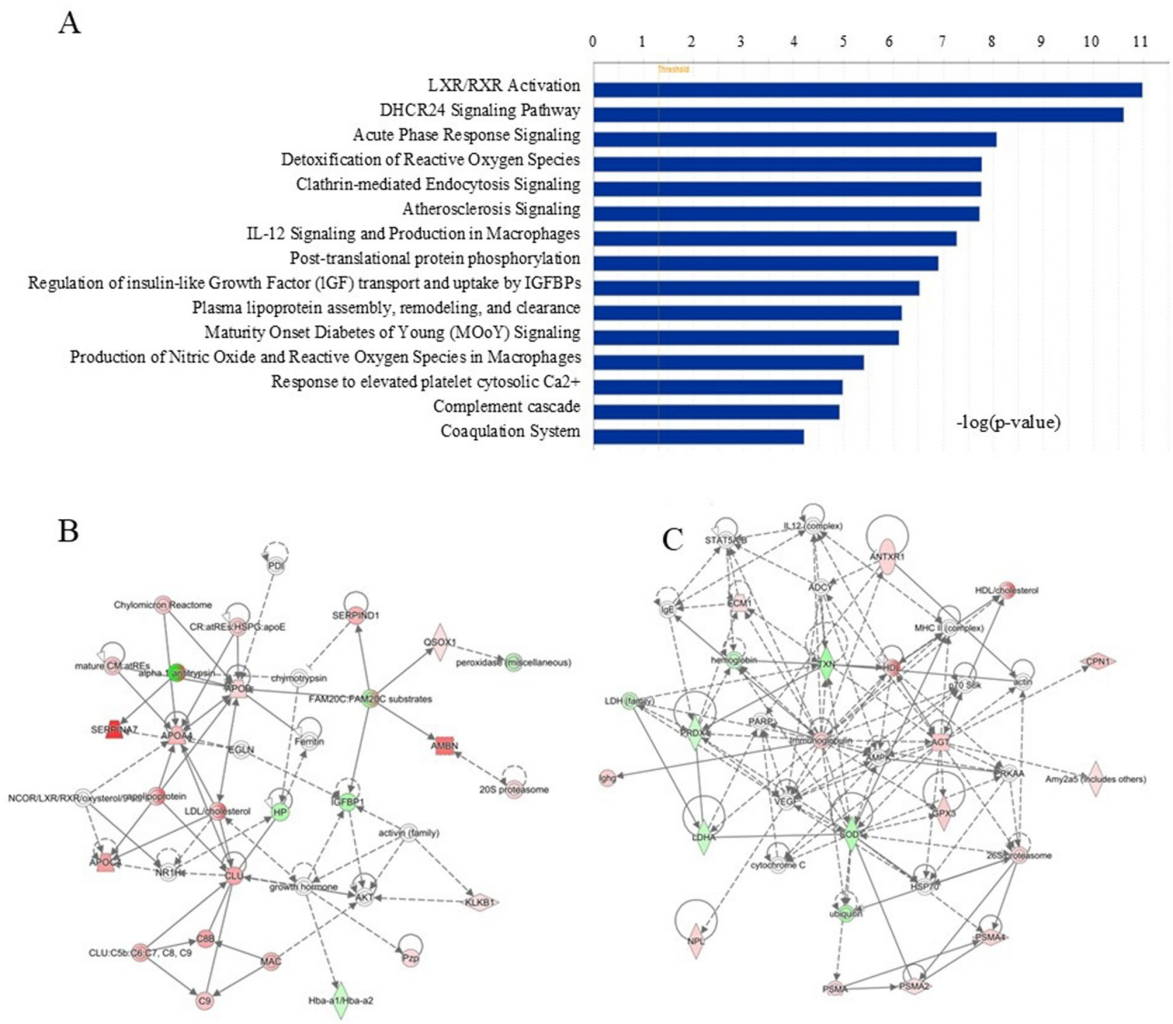
Gene networks of biological functions and/or diseases in IPA. **(A)** Top enriched pathways for the 59 significantly changed proteins according to the IPA. **(B)** Cardiac Dysfunction, Cardiovascular Disease, Neurological Disease (E Score = 35). The gene of the most important proteins included C8b, Apoa4, Clu, C9, Ambn, Serpind1 Hcf2, Qsox1 Qscn6 Sox2, Klkb1 Klk3 Pk. **(C)** Free Radical Scavenging, Hereditary Disorder, Small Molecule Biochemistry (E Score = 32). The gene of the most important proteins included Antxr1, Ecm1, Txn Txn1, Cpn1, Prdx1, Agt Serpina8, Amy2, Gpx3, Sod1, Psma2, Psma4, Npl.

Based on biological functions and/or diseases in the Ingenuity Pathway Knowledge Base, gene networks were analyzed using the IPA software. The most enriched pathways in the 59 changed proteins are shown in Figure 2A. In total, fifteen pathways were found by differentially expressed proteins, and the top five pathways according to their significance were LXR/RXR Activation, DHCR24 Signaling Pathway, Acute Phase Response Signaling, Detoxification of Reactive Oxygen Species, and Clathrin-mediated Endocytosis Signaling. Two networks were identified. Cardiac Dysfunction, Cardiovascular Disease, Neurological Disease (E Score=35) in Figure 2B and Free Radical Scavenging, Hereditary Disorder, Small Molecule Biochemistry (E Score=32) in Figure 2C. Both of the two networks have never been reported before.

The developmental process of the body was investigated in this experiment. Using the IPA software, more than 10 categories related to the development and functions were enriched (Table 5).

**Table 5:** Enrichment of diseases or functions annotation in IPA analysis.

**Categories**	**Diseases or functions annotation**	***p*-value**	**Molecules**
Hematological system development and function, tissue morphology	Quantity of macrophages	2.89E-07	9
Hematological system development and function, tissue morphology	Quantity of antigen presenting cells	3.09E-08	11
Hematological system development and function, tissue morphology	Quantity ot myeloid cells	8.79E-09	15
Tissue development	Formation of extracellular matrix	2.46E-09	6
Hermatological system development and function, tissue morphology	Quantity of blood cells	5.80E-07	17
Hematological system development and function	Hemostasis	6.60E-07	8
Cellular movement, hematological system development and function	Cell movement of leukocytes	1.22E-07	16
Cellular development, organ development, skeletal and muscular	Proliferation of smooth muscle cells	3.07E-07	9
Cellular development, organ development, skeletal and muscular	Proliferation of muscle cells	1.33E-07	11
Cellular movement, hematological system development and function	Cell movement of neutrophils	3.27E-07	10
Tissue morphology	Quantity of cells	4.57E-07	23
Cellular movement, hematological system development and function	Cell movement of phagocytes	4.32E-07	13
Hematological system development and function, nervous system development and function	Quantity of microglia	1.32E-07	5
Cardiovascular system development and function, organismal development	Angiogenesis	2.65E-07	17

## 4 Discussion

In our study, six individual plasma samples from FW and NB rats were analyzed using LC-MS/MS. A total of 639 proteins were identified in the serum of newborn rats and these proteins were associated with specific functions (Supplementary material). The changes that occurred within a week after birth were focused. This was the first time that plasma proteomics has been used to describe the early life development of animals. By performing principal component analysis and heat map analysis on the identified proteins, it can be clearly seen that plasma proteomics can not only distinguish between the two periods well, but also show individual differences well.

In the experiment, 59 proteins were showed significant differences, and some of them changed more than 10 times in abundance (Table 2). Comparing our data with published human studies, we found that 15 proteins have been reported (Table 3), and there were more differentially expressed proteins identified for the first time. This would enrich understanding of the early development of animals. Thyroxine-binding globulin, significant elevations in both rat and human studies, is the major thyroid hormone transport protein in serum. Thyroid hormone promotes metabolism and growth and development, and is essential for the development and growth of long bones, brain and reproductive organs. Thyroxine-binding globulin might play an important role in this biological process; Ameloblastin, which was involved in the mineralization and structural organization of enamel, implicated in cell signaling and polarity, cell adhesion to the developing enamel matrix, and stabilization of prismatic enamel morphology (24). Carboxylesterase 1C was responsible for the detoxification of a wide range of drugs and xenobiotics, and might contribute to cholesterol, fatty acid and lung surfactant metabolism (25). In our study this might be related to the development of the lungs. Glutathione S-transferase omega-1, a member of the glutathione transferase superfamily (GSTs) involved in the modulation of cell survival, proliferation and metabolism (26), was present in chemosensory tissues and fluids of the nasal/oral cavities where they protect tissues from exogenous compounds, including food molecules (27), we speculated that this might be related to newborn rat starting to eat. Complement component C8 was one constituent of the membrane attack complex (MAC) that plays a key role in the innate and adaptive immune response by forming pores in the plasma membrane of target cells. This might be related to the development of the complement system. Apolipoprotein D and Apolipoprotein C-IV were both increased significantly. Apolipoprotein D as a component of lipoproteins implicated in lipid metabolism was expressed associated with aging and in the presence of neuropathological processes (28), Apolipoprotein C-IV might participate in lipoprotein metabolism. Clusterin, a glycosylated protein with multiple biological functions, has attracted extensive research attention (29), it significantly increased both in our study and other human studies. Coagulation factor VII could initiate the extrinsic pathway of blood coagulation, it could be seen from the experiment that with the development of neonatal rats, coagulation function is constantly enhanced. Corticosteroid-binding globulin, as a major transport protein for glucocorticoids and progestins in the blood of almost all vertebrate species (30), had structure-function implications from species differences (31). These protein molecules were involved in important biological processes and played important roles. Our study described the changes of these proteins during development, suggesting that they were closely related to developmental processes. The changes of many of these proteins were reported for the first time, providing clues for further study on the new functions and related mechanisms of proteins.

Compared to other studies, many proteins showed similar changes; however, some differed. Angiotensinogen levels increased in both the current study and our previous study, but not in other studies (3, 16, 32). Haptoglobin level also differed from that in our previous study, and the reason for this discrepancy is currently unclear. In our study, insulin-like growth factor (IGF)-binding proteins (IGFBP) decreased significantly, and these changes were different in two other experiments (3, 16). IGFBP-1 and IGFBP-2 have high levels of circulation in the fetus during gestation and decrease with growth, whereas IGFBP-3 becomes the main carrier of IGFs in the serum (33). Nevertheless, additional protein changes have not been reported, which supplements previous data and further enriches our understanding of early development in newborn rats. In our experiment, P value was corrected by BH method for significantly differential proteins. At the same time, many proteins with uncorrected *P* value < 0.05 were observed (Supplementary material), and some of them were also reported in other literature, such as Complement C3, the levels of complement 3 were increased in the first week after birth (3), Compared to our previous study, complement 3 levels also increased during the development of fetal rats (34). These differentially expressed proteins need to be further studied.

An analysis of Gene Ontology (GO) categories revealed four biological processes related to oxidative metabolism: hydrogen peroxide catabolism, cellular oxidant detoxification, removal of superoxide radicals, and response to hydrogen peroxide. This is likely linked to environmental changes experienced by newborn rats after birth. Many proteins participate in the innate response and classical pathway of complement activation. This is similar to that in the first week of human life (16). This indicated that the immune systems of rats and humans share similarities during early development. Because the complement system is a rapid and effective immune surveillance system that contributes greatly to physiological homeostasis by eliminating cell debris and infectious microorganisms (35), these changes make young neonates more suitable for survival.

Based on ingenuity pathway analysis, 12 pathways were enriched. Many pathways coincide with those revealed in human studies (3, 4). This suggests that NB rats may undergo vigorous material metabolism in the early stages of development and that rats and humans undergo many similar changes during their growth. Interestingly, using the IPA software, more than 10 categories related to the development and functions were enriched (Table 5), and we speculated that these proteins were involved in the development of early vital organs in rats. These molecules changed in organs or tissues at different developmental stages could serve as potential markers for evaluating the developmental process of the body or diseases related to development.

In our study, some specimens were from different pregnant rats, and compared with NB rats from the same mother, large differences were observed. This may be due to individual differences. Even twins have shown a large variability in protein abundance of the quantitative proteome of plasma proteins (36).

In this study, the changes of plasma proteome in rats during two different periods were studied. Six individual plasma samples at each time point were studied in total; and this sample number was small. Proteins with fold change >2 and p.adjust.BH < 0.05 were considered as significant differential proteins. To minimize the variation in external factors, we carefully controlled the selection of subjects in our study by including standardized blood collection, processing, and storage protocols.

The sample size should be increased in order to get better experimental conclusions in future studies with technology develops. Owing to the limited experimental conditions, this study did not remove the high abundance protein in plasma, nor did it select more time points for research. In future research, richer and more detailed findings, such as a higher monitoring of organ or phylogeny, are expected to be found if the high abundance proteins are removed and the detection time points are increased.

## 5 Conclusion

This is the first study analyzing early mammalian development using liquid chromatography-mass spectrometry proteomics technology. Most of the changes in rat plasma proteome are similar as those in humans, and we also found some unreported changes, such as network of Cardiac Dysfunction, Cardiovascular Disease, Neurological Disease and Free Radical Scavenging, Hereditary Disorder, Small Molecule Biochemistry. Many protein molecules are involved in over 10 categories related to the development and functions. These molecules changed in organs or tissues at different developmental stages and could serve as potential markers for evaluating the developmental process of the body.

## Data Availability

The datasets presented in this study can be found in online repositories. The names of the repository/repositories and accession number(s) can be found in the article/Supplementary material.
