# Supplementary material for: Changes in rat plasma proteomes during the first week after birth
Source: Front Vet Sci. 2025 Feb 25;12:1440716. doi: 10.3389/fvets.2025.1440716 (PMC11894578; doi:10.3389/fvets.2025.1440716)
Supplement: Supplementary file 1 [file Table_1.docx]

**Table S1. Significantly differentiated proteins in plasma proteome of FW rats vs NB rats**

| **Gene Names** | **Uniprot Accession** | **Protein names** | **p.adjust.BH** | **FC** | **AUC** |
| --- | --- | --- | --- | --- | --- |
| Minpp1 | O35217 | Multiple inositol polyphosphate phosphatase 1 | 0.029664 | 4.18 | 1.00 |
| Amy2 | P00689 | Pancreatic alpha-amylase | 0.029929 | 4.06 | 1.00 |
| Agt Serpina8 | P01015 | Angiotensinogen | 0.016214 | 6.91 | 1.00 |
| Col1a2 | P02466 | Collagen alpha-2(I) chain | 0.010063 | 5.19 | 1.00 |
| Apoe | P02650 | Apolipoprotein E (Apo-E) | 0.039459 | 6.69 | 0.94 |
| Apoa4 | P02651 | Apolipoprotein A-IV | 0.006463 | 9.05 | 1.00 |
| Clu | P05371 | Clusterin | 0.016198 | 11.37 | 1.00 |
| C4 C4a | P08649 | Complement C4 | 0.021423 | 4.53 | 0.97 |
| Ces1c Es2 | P10959 | Carboxylesterase 1C | 0.028532 | 16.44 | 0.94 |
| Klkb1 Klk3 Pk | P14272 | Plasma kallikrein | 0.039064 | 3.94 | 0.94 |
| RT1-Aw2 | P15978 | Class I histocompatibility antigen | 0.029664 | 5.40 | 0.97 |
| Fuca1 Fuca | P17164 | Tissue alpha-L-fucosidase | 0.042828 | 6.27 | 0.94 |
| Psma2 | P17220 | Proteasome subunit alpha type-2 | 0.049802 | 4.30 | 0.89 |
|  | P20759 | Ig gamma-1 chain C region | 0.01085 | 6.41 | 1.00 |
| Psma4 | P21670 | Proteasome subunit alpha type-4 | 0.046792 | 5.75 | 0.94 |
| Apod | P23593 | Apolipoprotein D | 0.010176 | 24.97 | 1.00 |
| Gpx3 | P23764 | Glutathione peroxidase 3 | 0.013026 | 6.40 | 1.00 |
| Serpina7 Tbg | P35577 | Thyroxine-binding globulin | 0.002375 | 26.27 | 1.00 |
| Afm | P36953 | Afamin (Alpha-albumin) | 0.002375 | 8.52 | 1.00 |
| Rpl13 | P41123 | Large ribosomal subunit protein eL13 | 0.01709 | 9.63 | 1.00 |
| Itgb1 | P49134 | Integrin beta-1 | 0.023806 | 8.20 | 0.97 |
| C8b | P55314 | Complement component C8 beta chain | 0.045883 | 11.91 | 0.94 |
| Apoc4 Ecl | P55797 | Apolipoprotein C-IV | 0.022794 | 12.74 | 0.97 |
| Antxr1 | Q0PMD2 | Anthrax toxin receptor 1 | 0.013613 | 5.31 | 1.00 |
| Ccndbp1 | Q5BK06 | Cyclin-D1-binding protein 1 | 0.046005 | 4.09 | 0.92 |
| Ambn | Q62840 | Ameloblastin | 0.005244 | 18.51 | 1.00 |
| Ecm1 | Q62894 | Extracellular matrix protein 1 | 0.016214 | 4.52 | 1.00 |
| C9 | Q62930 | Complement component C9 | 0.039064 | 7.87 | 1.00 |
| A1m Pzp | Q63041 | Alpha-1-macroglobulin | 0.045588 | 5.03 | 0.94 |
| Itih3 | Q63416 | Inter-alpha-trypsin inhibitor heavy chain H3 | 0.042528 | 4.52 | 1.00 |
| Azgp1 | Q63678 | Zinc-alpha-2-glycoprotein | 0.045588 | 4.53 | 0.97 |
| Serpind1 Hcf2 | Q64268 | Heparin cofactor 2 | 0.013843 | 9.67 | 1.00 |
| Ptprs | Q64605 | Receptor-type tyrosine-protein phosphatase S | 0.004524 | 3.32 | 1.00 |
| Npl | Q66H59 | N-acetylneuraminate lyase | 0.022423 | 5.69 | 0.97 |
| Cpq Pgcp | Q6IRK9 | Carboxypeptidase Q | 0.029664 | 4.98 | 0.94 |
| Qsox1 Qscn6 Sox2 | Q6IUU3 | Sulfhydryl oxidase 1 | 0.043833 | 3.68 | 1.00 |
| Apob Aa1064 Ac1-060 | Q7TMA5 | Apolipoprotein B-100 | 0.013026 | 5.59 | 1.00 |
| Serpina11 Ab1-046 | Q7TPA5 | Serpin A11 | 0.045883 | 3.85 | 0.97 |
| F7 | Q8K3U6 | Coagulation factor VII | 0.018177 | 10.71 | 1.00 |
| Cpn1 | Q9EQV8 | Carboxypeptidase N catalytic chain | 0.01782 | 7.79 | 1.00 |
| Adam15 Mdc15 | Q9QYV0 | Disintegrin and metalloproteinase domain-containing protein 15 | 0.035126 | 4.39 | 0.97 |
| Gsto1 | Q9Z339 | Glutathione S-transferase omega-1 | 0.045396 | 16.17 | 0.94 |
| Dlk1 Dlk Pref1 Zog | O70534 | Protein delta homolog 1 | 0.006711 | 0.30 | 1.00 |
| Hba1 Hba-a1 | P01946 | Hemoglobin subunit alpha-1/2 | 0.016214 | 0.42 | 1.00 |
| Ldha Ldh-1 Ldh1 | P04642 | L-lactate dehydrogenase A chain | 0.045533 | 0.39 | 0.94 |
| Rps21 | P05765 | Small ribosomal subunit protein eS21 | 0.029664 | 0.37 | 1.00 |
| Hp Ba1-647 | P06866 | Haptoglobin | 0.029929 | 0.28 | 1.00 |
| Sod1 | P07632 | Superoxide dismutase [Cu-Zn] | 0.013316 | 0.30 | 1.00 |
| Dbi | P11030 | Acyl-CoA-binding protein | 0.014853 | 0.31 | 1.00 |
| Txn Txn1 | P11232 | Thioredoxin | 0.016214 | 0.26 | 1.00 |
|  | P11517 | Hemoglobin subunit beta-2 | 0.009543 | 0.29 | 1.00 |
| Igfbp1 Igfbp-1 | P21743 | Insulin-like growth factor-binding protein 1 | 0.014853 | 0.26 | 0.97 |
| Serpina6 Cbg | P31211 | Corticosteroid-binding globulin | 0.002375 | 0.10 | 1.00 |
| Prdx2 Tdpx1 | P35704 | Peroxiredoxin-2 | 0.01709 | 0.31 | 0.94 |
| Fabp5 | P55053 | Fatty acid-binding protein 5 | 0.006463 | 0.39 | 1.00 |
| Rps27a Uba80 Ubcep1 | P62982 | Ubiquitin-ribosomal protein eS31 fusion protein | 0.006711 | 0.30 | 1.00 |
| Prdx1 Tdpx2 | Q63716 | Peroxiredoxin-1 | 0.002375 | 0.42 | 1.00 |
| Hamp Hepc | Q99MH3 | Hepcidin | 0.049895 | 0.28 | 0.92 |
| Ccn3 Nov | Q9QZQ5 | CCN family member 3 | 0.029664 | 0.26 | 1.00 |
|  |  |  |  |  |  |
